# Evaluating Biogenicity on the Geological Record With Synchrotron-Based Techniques

**DOI:** 10.3389/fmicb.2019.02358

**Published:** 2019-10-11

**Authors:** Flavia Callefo, Lara Maldanis, Verônica C. Teixeira, Rodrigo Adrián de Oliveira Abans, Thiago Monfredini, Fabio Rodrigues, Douglas Galante

**Affiliations:** ^1^Brazilian Synchrotron Light Laboratory, Brazilian Center for Research in Energy and Materials, Campinas, Brazil; ^2^Institute of Physics of São Carlos, University of São Paulo, São Paulo, Brazil; ^3^Interunits Graduate Program in Biotechnology, University of São Paulo, São Paulo, Brazil; ^4^Fundamental Chemistry Department, University of São Paulo, São Paulo, Brazil

**Keywords:** synchrotron, biogenicity, spectroscopy, imaging, biostructures

## Abstract

The biogenicity problem of geological materials is one of the most challenging ones in the field of paleo and astrobiology. As one goes deeper in time, the traces of life become feeble and ambiguous, blending with the surrounding geology. Well-preserved metasedimentary rocks from the Archaean are relatively rare, and in very few cases contain structures resembling biological traces or fossils. These putative biosignatures have been studied for decades and many biogenicity criteria have been developed, but there is still no consensus for many of the proposed structures. Synchrotron-based techniques, especially on new generation sources, have the potential for contributing to this field of research, providing high sensitivity and resolution that can be advantageous for different scientific problems. Exploring the X-ray and matter interactions on a range of geological materials can provide insights on morphology, elemental composition, oxidation states, crystalline structure, magnetic properties, and others, which can measurably contribute to the investigation of biogenicity of putative biosignatures. Here, we provide an overview of selected synchrotron-based techniques that have the potential to be applied in different types of questions on the study of biosignatures preserved in the geological record. The development of 3rd and recently 4th generation synchrotron sources will favor a deeper understanding of the earliest records of life on Earth and also bring up potential analytical approaches to be applied for the search of biosignatures in meteorites and samples returned from Mars in the near future.

## Introduction

Elucidating the origin and evolution of life on Earth, as well as the possibilities of its occurrence and preservation outside the planet are topics of primordial interest to the astrobiological community. For the search of life in extraterrestrial worlds, distinguishing and understanding the preservation of the earliest records of life on Earth is a step of fundamental importance. Although evidences of life have been reported in rocks with more than 3.7 billion years (Ohtomo et al., 2014), several descriptions of very ancient chemical or morphological biosignatures have been debated by the scientific community over the past decades (Brocks et al., 1999; Brasier et al., 2015; Nutman et al., 2016; Dodd et al., 2017; Schopf et al., 2018). The challenge becomes even greater when the putative biosignatures are from outside Earth (whether in meteorites, or chemical or morphological signals measured *in situ* or that will be collected in future missions to Mars). The Viking missions on the 1970’s found chemical traces that could be related to metabolism on the surface of Mars (Levin and Straat, 1977), which were later contested. The same happened in the 1990’s, when McKay et al. (1996) described supposed biological microscopic structures on the ALH84001 meteorite. More recently, Noffke (2015) presented sedimentary structures resembling terrestrial microbially-induced sedimentary structures (MISS) on Mars, based on morphological analysis of images from the rover Curiosity, which instigated the astrobiological community in the hope of finding chemical biosignatures or other evidences that could support their biogenicity.

The application of analytical techniques in paleo and astrobiology has contributed to the advancement of knowledge about the indicatives of past or present biological activity from macroscopic to nanometric scales. Techniques such as Raman Spectroscopy, X-ray Diffraction, X-ray Tomography, Electron Microscopy and nanoscale secondary ion mass spectrometry (Nano SIMS) are now being routinely applied on Earth sciences. Meanwhile, techniques based on synchrotron light are gaining more space as promising tools for the non-destructive investigation of paleobiological samples, giving rise to an emerging field known as paleometry (Gomes et al., 2019). Some of these techniques are summarized in Table 1.

**TABLE 1:** List of selected synchrotron-based techniques for assessing scientific characters related to biogenicity.

**Synchrotron technique**	**Scientific character**	**Advantages**	**Disadvantages**	**References**
PXCT	Morphology in 3D	Image whole microorganisms non-destructively with nanometric resolution	Time-consuming	Cunningham et al., 2015; Guizar-Sicairos et al., 2015
XRF	Elemental mapping	Flexible sampling are and resolution. Allows 2D and 3D mappings. High sensitivity	Elements detected limited to the energy range available	Sforna et al., 2016; Marshall et al., 2017; Allwood et al., 2018; Callefo et al., 2019
XAS/STXM	Elemental speciation, fingerprinting	Allows chemical speciation for discriminating bio-elements/STXM – Soft X-rays decrease the risk of damage by radiation for organic matter; can be performed *in situ* on thin samples; do not need further preparation, such as extraction from the bulk samples	Depends on standards for fingerprint or fundamental calculations	Lemelle et al., 2008; Bernard et al., 2009; De Gregorio et al., 2009; Alleon et al., 2016; Grosch et al., 2017; Rodelli et al., 2018; Sancho-Tomás et al., 2018
XMCD	Magnetic behavior	Directly probes the magnetic states of minerals	Relies on low energy beamlines, under ultra-high vacuum	Coker et al., 2007; Carvallo et al., 2008
XRD	Crystalline structure	Measure the 3D organization of crystalline structures of minerals, even for dilute phases; On 4th generation machines it will be minimally invasive	Sample preparation can be destructive	Thomas-Keprta et al., 2000; Tadic and Epple, 2004; Miot et al., 2014; Che et al., 2016; Iñiguez et al., 2017; Till et al., 2017
XEOL	Optical properties	Measure the optical emission after X-ray excitation, carrying information of the electronic structure and defects. Useful for many minerals, similar to cathodoluminescence	Not all minerals will luminesce on the given condition; it has to be combined to other techniques for a complete interpretation of data	Kolodny et al., 1996; Rakovan and Reeder, 1996; Dartnell and Patel, 2014; Gaft et al., 2015; Lin et al., 2015; Marshall et al., 2017; Shkolyar et al., 2018

*Advantages and disadvantages in comparison with conventional techniques and examples of applicability are also shown.*

Synchrotron accelerators produce light with unique qualities, such as high brilliance (flux of photons *per* unit area), broad, continuous energy range, and high coherence, which are unattainable by conventional light sources, such as lasers, lamps or X-ray tubes. These characteristics allow the use of multiple physical phenomena to unveil the original elemental and molecular composition, mineralogy and morphology of samples, with higher sensitivity, penetrability and resolution than conventional techniques applied for the same purposes. In the present review, we will discuss recent applications and potentials of some synchrotron-based techniques for evaluating biosignatures in the geological record.

## Biosignatures and Implications

By definition, biosignatures are any signs, objects, substances, and/or patterns generated by biological activity (Des Marais et al., 2003). The definition of biosignature, its applicability for exoplanets and how it can be used as analogs for the search of life outside Earth have been the focus of several studies such as White (1974), Walsh (1992), Schopf (1993), Mojzsis et al. (1996), Des Marais and Walter (1999), Brocks et al. (1999), Westall et al. (2000), Banfield et al. (2001), Javaux et al. (2001), Des Marais et al. (2002), Gorbushina et al. (2002), Thomas-Keprta et al. (2002), van Zuilen et al. (2002), Garcia-Ruiz et al. (2003), Lindsay et al. (2005), Douglas et al. (2008), Summons et al. (2008), Westall (2008), Westall (2009), Westall and Cavalazzi (2011), Cockell (2014), Brasier et al. (2015), Meadows (2017), Catling et al. (2018), Meadows et al. (2018), Schwieterman et al. (2018), Walker et al. (2018), Westall et al. (2019), among several others.

Biosignatures include, among others, (i) morphological features, such as preserved cells or extracellular components, as fossilized extracellular polymeric substance (EPS); (ii) biogenic minerals, such as those biologically-induced (i.e., biogenic dolomite precipitated in microbialites and microbial mats) or biologically-controlled (i.e., endoskeletons and hard mineralized parts of metazoans), which sometimes present crystallographic characteristics and/or physical properties that can be distinguished for those abiotic minerals (i.e., biogenic magnetite, such as magnetites produced inside the cells of magnetotactic bacteria (magnetosomes); (iii) specific textures or biogenic fabrics in rocks originated by microbial activity, such as fenestral fabrics in microbialites; (iv) molecular vestiges linked to biological activity, such as organic ligands, lipids, organic macromolecules, etc; (v) specific chemical characteristics of bioprocessing, such as chirality in organic molecules; (vi) stable isotopes favorable to biogenic patterns and bioprocessing. Interpreting these signals as biosignatures is not always trivial, especially if these samples are from deep time context (Javaux, 2019). One example is the distribution pattern of bio-elements (trace or major) in the samples of interest. The interpretation of signals of past life in these distributions may be complex and others aspects need to be considered, such as the co-occurrence of other types of biosignatures, such as morphological and chemical (isotopes, co-localization of elements, combinations with organic signatures, etc.), or characteristics of the authigenic mineralogy and properties of the original geological context.

The complexity of interpretation also increase considering the possibilities of posterior contaminations and the several changes in the original characteristics of mineralogy and geological features, the action of metamorphism and changes of temperature which can degrade or modify the organic biosignatures, besides several other alterations during the post-burial processes over time. Thus, it is important to do critical interpretations of the signs of early life in order to avoid misinterpretations and/or equivocal biosignatures. There are several examples in the literature regarding the contestation or reassessment of alleged evidence of ancient life on Earth and outside it (McKay et al., 1996; Golden et al., 2000; Thomas-Keprta et al., 2002, 2009; Steele et al., 2012). Some will be discussed in the following specific sessions.

## Imaging Morphological Biosignatures

Morphological traces of microorganisms are important biosignatures for providing direct insights into ancient life ultrastructure, evolution, paleo-environment and also to help to understand the physico-chemical conditions that allowed their long-term preservation. High resolution microscopies applied so far, such as Scanning Electron Microscopy (SEM) or Transmission Electron Microscopy (TEM) have allowed significative advances on the understanding of the earliest fossilized microbes. Their application, however, is limited by the low penetration depth of electrons, thus requiring destructive sample preparation for exposing the structures from within the rock matrix (potentially introducing artifacts), or by preparing ultra thin (<100 nm) sections that can sample only a small fraction of the specimen. 3D images can be achieved by using a focused ion beam (FIB) coupled to a SEM (FIB-SEM), and combining sequential milling with concurrent Energy Dispersisve Spectroscopy (EDS-SEM) imaging. Although being a destructive approach, it has allowed even some of the most ancient microfossils to be investigated at the nanoscale, providing insights into their chemistry, ultrastructure, taphonomy and investigating the biogenicity of these structures (Wacey et al., 2012; Brasier et al., 2015).

X-rays can penetrate objects and provide information of their interior non-destructively, therefore having the potential of overcoming the limitations of electron microscopy. Conventional X-ray imaging, such as the widely known Computed Tomography (CT), is based in the absorption of X-rays, a physical interaction which relies on the densities and/or atomic numbers of the materials comprising the specimens. For mineralized structures such as some fossils, this can represent a limitation in contrast. Synchrotron sources allow other contrast modalities to be explored, such as the phase contrast. Phase-contrast μ-CT has become critical for paleobiological studies in the last decades (Tafforeau et al., 2006; Cunningham et al., 2012; Maldanis et al., 2016), due to its capacity of extracting 3D information from these homogeneously dense specimens non-invasively, revealing even the preservation of soft tissues. Recently, the limit of resolution of X-ray microscopy has been pushed forward by the development of techniques based on Coherent Diffraction Imaging (CDI), specially Ptychography. This lensless method allows specimens of tens of microns to be imaged with nanometric resolution, and can also be applied in 3D, receiving the name of Ptychographic X-Ray Computed Tomography, or PXCT (Holler et al., 2014). This imaging approach is based in the collection in the far-field of diffraction patterns partially superposed while scanning the sample. This redundancy of measurements allows the reconstruction of the specimen’s complex refraction index (both absorption and phase components) using iterative algorithms instead of X-ray lenses. PXCT has been only briefly applied to the study of microfossils (Cunningham et al., 2015; Guizar-Sicairos et al., 2015), and its potential for evaluating the biogenicity of morphological biosignatures has still to be further explored. Nevertheless, its potential for unveiling whole fossilized microbes within their geological context also configures it as a potential methodology to be used for searching morphological biosignatures potentially preserved in rocks returned from Mars in the near-future.

## Elemental Mapping of Chemical Biosignatures

Metabolic processes of biological systems can generate traces or patterns uncommon to abiotic systems. For geobiological materials, these biosignatures can be present in the form of biominerals with different crystalline organizations or specific distributions, mineral assemblages in association with organic matter and modifications on the mineral surface (Banfield et al., 2001). Some elements are considered to be bio-essential and bio-functional (i.e., P, S, Ti, V, Cr, Mn, Fe, Co, Ni, Cu, Zn, Mo, As, and Pb) (Williams and Frausto da Silva, 1996), and when co-localized and/or associated to specific morphological patterns, such as layered distribution or in consonance with organic matter, they could represent biosignatures, including results of biological processes. Moreover, the geochemical composition and growth processes of microbial mats favor the binding of some metals, which can be reorganized during the development of the community, forming biominerals or being adsorbed on the surface of minerals.

Identifying and mapping the chemical elements present in geobiological structures can provide means of supporting their biogenicity and also understanding their ecological interactions and modes of preservation. Nevertheless, identifying these elements and distinguishing patterns generated by biotic from abiotic processes require approaches with high-spatial resolution, sensitivity to a broad range of elements and to trace element concentrations. In questions about biogenicity studies, in particular for elemental distribution analyzes such as X-ray fluorescence mapping, it is important to consider the co-location of elements with morphological structures of interest, original lithology and its possible changes, the co-occurrence of biogeochemical elements of interest and also their abundance limits taking into account the geological and preservational context. The combination of as much evidence as possible that can explore the depositional history, and especially the diagenetic alterations that may have exerted any changes in the preservation of authigenic characteristics, it is important to minimize any misinterpretation, especially in deep time rocks.

**FIGURE 1:**
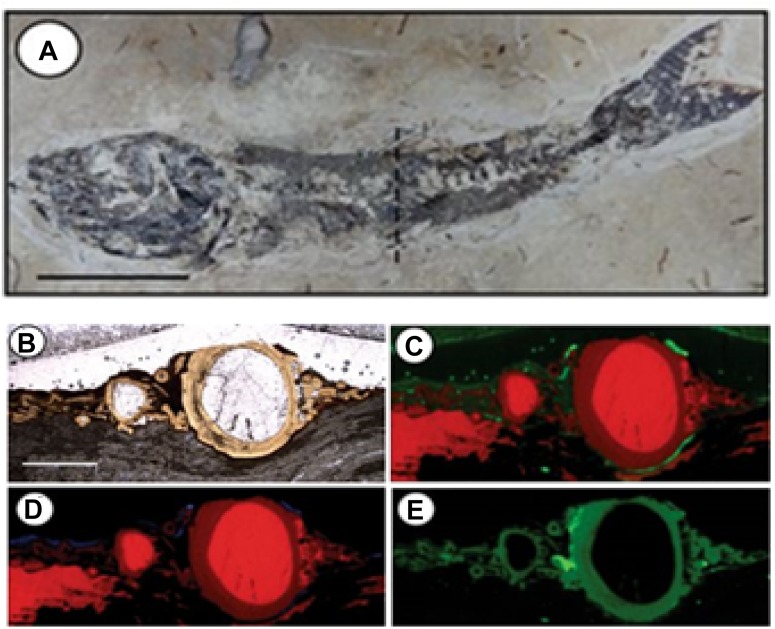
Application of synchrotron-based X-ray microfluorescence in fossil fish from Crato Member, Brazil (Osés et al., 2017), showing the potential of elemental mapping in providing information about the original elemental composition (interpreted as biosignatures) and diagenetic processes (interpreted as secondary incorporation). In this case, the authors used the elemental mapping to identify some metals (i.e., Fe, Cu, and Zn) related to the incorporation by microbial activity, in order to discuss the biogenicity of pyrites and understand the role of bacteria in the tafonomical process. **(A)** Mapped specimen (mapped area in a cross section in the dashed line), scale: 1.5 cm; **(B)** area mapped by X-ray microfluorescence, scale: 2 mm; **(C)** Ca-red, Fe-green; **(D)** Ca-red, Cu-blue; **(E)** Zn (modified from Osés et al., 2017). Reprinted by permission from Scientific Reports (open access), Creative Commons CC BY, Copyright (2017), Springer Nature.

Synchrotron-based X-ray fluorescence (SR-XRF) allows the identification, mapping and semi-quantification of chemical elements even in concentrations of parts per billion (ppb), and has a spatial resolution primarily dependent on the size of the X-ray beam, which, in the case of the new generation synchrotrons, can reach nanometric dimensions. The range of elements that can be detected with this approach depends on the energy of the X-rays, once the fluorescence phenomenon relies on the removal of strongly-binded inner-shell electrons, creating vacancies that need to be filled by external electrons. The difference of energy between the electrons involved in this process is emitted as photons, generating a fluorescence pattern which is specific to each element. One example of case study using SR-μ-XRF was applied to discuss the biogenicity of pyrites in a microbial context in order to understand the role of bacteria in the tafonomical process of well-preserved fossilized organisms in Crato Basin, Brazil (Osés et al., 2017). The authors combined morphological biosignatures, such as putative fossilized bacterial EPS observed in SEM with framboidal pyrites, using the SR-μ-XRF mapping to identify some metals (i.e., Fe, Cu, and Zn) which could be incorporated in the system by microbial activity (Figure 1).

Sforna et al. (2016) used SR-μXRF and complementary approaches to assess the metal incorporation in living microbialites and their remobilization during a simulated diagenetic processes. They mapped the distribution of metals triggered by secondary abiotic processes and provided a basis of comparison for evaluating ancient Precambrian microbialites. Recently, SR-μ-XRF was also applied in combination with conventional techniques and magnetic analysis by Callefo et al. (2019), in order to evaluate the biogenicity of iron minerals in carboniferous rhythmites (periodic sedimentary depositions). The distribution pattern of iron in co-occurrence with putative MISS and organic matter, plus the magnetic signal compatible with biogenic magnetite, indicated a biological origin for the iron minerals, allowing a reassessment the depositional history of the geological site.

Studying organic-walled microfossils, Marshall et al. (2017) reported the application of XRF for mapping V, an element present in chlorophyll and heme porphyrin pigments. The authors propose that the co-localization of this element within microfossil-like morphologies and carbonaceous composition can be used as a biosignature for putative microfossils, and could also be applied in samples returned from Mars in the near future.

**FIGURE 2:**
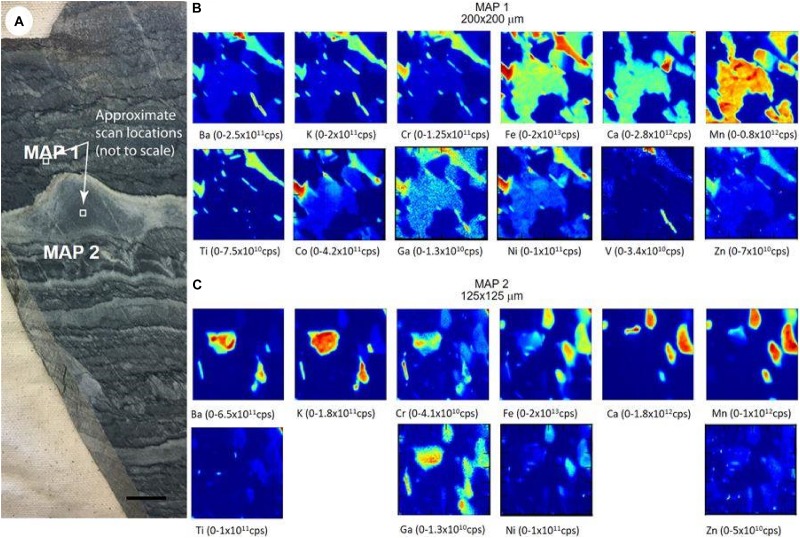
Distribution of trace elements in putative microbialites from 3.7 Ga Isua supracrustal belt, Greenland, originally published by Nutman et al. (2016) as putative stromatolites. The elemental maps were made with the application of synchrotron-based X-ray fluorescence, and the elemental maps makes part of the discussion of a plausible non-biological origin of the structures. **(A)** Photograph of the sample with the mapped areas (white squares, scale: 10 mm); **(B)** map of area 1; **(C)** map of area 2. For both maps the blue shows the highest X-ray intensity while the red shows the lowest intensity (counts per second); the maps are for K-shell X-rays except for Ba, which was detected using L-shell X-rays (Allwood et al., 2018). Reprinted by permission from Springer Nature.

Allwood et al. (2018), in order to reassess the biogenicity of putative microbialites from 3.7 Ga Isua supracrustal belt published by Nutman et al. (2016), used the distribution of trace elements relative to minerals in the putative structures (Figure 2). In combination of other techniques to reveal the three-dimensional, morphology and orientation of the structures, the X-ray fluorescence showed the distribution of major and trace elements inside the morphology of the putative stromatolites, revealing an alternative explanation for the origin of the structures. For the authors, it is plausible a non-biological formation of the structures, consisting of a feature caused after burial process by deformations in a carbonate-altered metasediments. The authors argue that in the previous interpretation (from Nutman et al., 2016) for the trace-elements in the three-dimensional structure as evidence of primary marine carbonate sedimentation, in fact was result as a mixture of samples, including micas, not only dolomite. Once micas can carry oligoelements in these rocks along the geological history, the biogenicity of the structures become questionable. This case may illustrate the need for further analysis and other evidences to complement the use of elemental biosignatures.

## Differentiating Biotic and Abiotic Elements Species

One of the main challenges in the search of chemical biosignatures associated to microfossils and microbialites is the fact that the original biochemical components of microorganisms are degraded and altered over time, while other non-biogenic elements are incorporated in the course of diagenetic processes (Lemelle et al., 2008). The metabolic activity of microorganisms, however, generates redox heterogeneities, which, sometimes in association to organic compounds, can constitute biosignatures (Miot et al., 2014). The speciation of elements provides information about oxidation state and chemical neighboring, which can provide valuable insights into microorganism-minerals interactions and ancient metabolisms.

The possibility to tune the energy of X-rays in synchrotron sources allows the application of X-ray Absorption Spectroscopy (XAS), an approach based on the analysis of the absorption profile of a given element prior to the core electrons removal with the incidence of X-rays (the same phenomenon described above for the generation of the fluorescence signal). The different regions analyzed on the absorption spectra gives rise to two different techniques, X-ray Absorption Near Edge Structure (XANES), and Extended X-ray Absorption Fine Structure (EXAFS). These techniques are strongly sensitive to the oxidation state of the elements and can also provide information about the coordination of the absorbing ions. When associated to the small beams available at scanning X-ray microscopes, speciation maps with nanometric resolution can be achieved. These techniques have already been applied at lower concentrations and at less-than-perfect sample conditions (Newville, 2014), as the ones usually present in natural geological samples.

There are several examples of the use of these techniques as a complement both in reinforcing and in questioning the biogenicity of ancient microbialites and microfossils. Lemelle et al. (2008) found biomarkers related to organic S associated to microfossils by evaluating the sulfur absorption K-edge. Grosch et al. (2017) used XANES to question the biogenicity of 3.47 Ga filament-shaped titanite microtextures in early Archean samples from Barberton Greenstone Belt, South Africa, which was considered the oldest microbial trace-fossil on Earth. The authors used temperature maps combined with μ-XANES for Fe speciation profiles in chlorites present in metabasalts that contained the filaments. The data pointed to metamorphic constraints that indicated incompatibility with the biogenicity of the structures. In contrast, De Gregorio et al. (2009) used XANES with Scanning-Transmission X-ray Microscopy (STXM) and complementary techniques in order to reinforce the biogenicity of 3.5 Ga putative microfossils from Apex Chert, Western Australia. The authors found similar characteristics on these specimens with biogenic kerogen from the *ca*. 1.9 Ga Gunflint Formation. In this study, XANES provided information about the chemical complexity of kerogen, which presented aromatic carbon and oxygenated functional groups. Sancho-Tomás et al. (2018) applied XANES and μ-XRF in combination with conventional analysis and showed the relationship between the biological processing of As with the mineralogy in recent hypersaline microbial mats, by evaluating the mineral occurrence, the As speciation and the elemental distribution.

With the intense photon beams of 3rd and 4th generation synchrotron sources, radiation damage of the samples should also be considered. Potentially preserved organic molecules are the most fragile ones, and can suffer from photooxidation and breakup depending on the measuring condition. However, even inorganic signatures can be altered, as X-rays can change the oxidation state of elements (e.g., photo-reduction of sulfur as reported by Moussallam et al., 2014), or produce defects on the crystal lattice of minerals. It is possible to use the advantage of the brilliant beams while minimizing exposition by performing very fast scans, both in energy (for spectroscopy) and in space (for imaging). This should be taken in consideration on the design of new beamlines that intend to be used on radiation-sensitive materials. Theoretical calculations and test measurements with standards can optimize the systems before the measurement, ir order to collect enough signal to allow the study, but delivering the minimum dose possible. Different strategies of analysis for mitigating and monitoring these effects for ancient materials have been reviewed by Bertrand et al. (2014).

It is possible to use the chemical information obtained with XAS as a contrast for an imaging approach, called STXM is a type of X-ray microscopy which uses XANES as its contrast mechanism (Ade and Urquhart, 2002). This approach works usually in the soft X-ray energy range (130–2.500 eV), and can reach nanometric scale of spatial resolution. This range of energy can interact with almost all elements, besides allow to map chemical species based on bonding structure. The use of soft X-rays also reduce the risks of damage for radiation, in comparison with the electron beam techniques (Lawrence et al., 2003). One advantage is the possibility of working with bulk samples, as long as it is transparent to the beam, and the possibility of imaging a number of key-elements of interest in the same sample. This technique has been utilized for determining the speciation of elements such as carbon and nitrogen in microfossils at submicrometric scale, such as organic microfossils from 1.88 Ga Gunflint Formation (Alleon et al., 2016), refining the knowledge about the degradation of organic biosignatures along the time, especially by the effects of temperature changes along the diagenetic processes.

Infrared techniques can also be powerful tools for studies of detection and study of the preservation or alteration of organic biosignatures. It possible to retrieve infrared spectra in absorption or emission of samples in gas, liquid or solid state, with high spectral resolutions and in a wide spectral range. Although there are several studies using conventional infrared techniques for the study of biosignatures (Guido et al., 2012; Preston et al., 2014; Gordon and Septhon, 2016; Gaboyer et al., 2017; Igisu et al., 2018; Stevens et al., 2019), there is still a lack of reference in the use of synchrotron-based FTIR (SR-FTIR) techniques for this purpose. The synchrotron-based approach has the advantage of the high flux and broad range of energy, which allows the acquisition of fast spectra with high signal-noise ratio. For biological signatures, this can mean a decrease in the risk of degradation of biosignatures during the measurement time.

An example of application of SR-FTIR on the evaluation of chemical biosignatures’ behavior during the fossilization process was presented by Benning et al. (2004), in which SR-FTIR micro-spectroscopy was applied to determine the response of the organic structure of live cyanobacterial cells. The vibration of the original components of the microbial cells (specific functional groups related to the cells), together with the characteristic vibrations for silica was analyzed during the progressive silicification. This is especially relevant, as silicification is one of the most common fossilization processes and present high capability of biosignature preservation during the geological time (Konhauser et al., 2004; Wacey et al., 2011; Campbell et al., 2015; Sugitani et al., 2015; Manning-Berg et al., 2019).

## Investigating the Structure, Optical and Magnetic Properties of Biominerals

The advantage of biominerals (Dove et al., 2003; Perry et al., 2007; Dupraz et al., 2009) in their use as biosignatures is due to they are more resistant to deep-time geological processes and to several alterations that can be caused by the diagenesis. In comparison with organic biomarkers, for instance, the biominerals are more persistent in nature over the time (Jimenez-Lopez et al., 2010). Biominerals are very important for the study of past life on the planet, as they can represent life records present in rocks of very old ages. In the case of magnetofossils, it can be a good biomarker for the presence of past life on Earth and beyond (McKay et al., 1996). However, in order to be able to use them as biosignatures, it is first necessary to know the intrinsic characteristics that differ them from minerals of abiotic origin, and to compare them in order to establish biogenicity parameters. Biominerals can be differentiated from minerals of abiotic origin due to some intrinsic characteristics that they present. It is known that the biomineralization processes can influence the organization of minerals, allowing one to differentiate biogenic and abiogenic minerals regarding, for instance, their crystalline structure and physical properties (Chang and Kirschvink, 1989; Bazylinski et al., 1995; Thomas-Keprta et al., 2000; Egli, 2004). In the biologically-controlled mineralization, the genetics of the organisms/microorganisms can control intrinsically the mineral nucleation according some physiological or morphological need (Mann, 2001; Dupraz et al., 2009). This intrinsic control can originate characteristics that can be distinguishable from those inorganic minerals, such as some properties and organizations observed in internal and external skeletons (i.e., Ma et al., 2016; Rao et al., 2016) or observed in different origins of magnetites (Bazylinski et al., 1995; Thomas-Keprta et al., 2000; Körnig et al., 2014). These biominerals (or organominerals, such as suggested by Mann (2001) when the mineral is genetically controlled during its formation) can be considered direct evidence of life, consisting, when properly detected, a consistent biosignature. For instance, there are at least six specific characteristics that can distinguish the intracellular magnetite from detrital magnetite (Thomas-Keprta et al., 2000). They are: small size of the crystals to until a few dozen nanometers (single domain – SD), controlled mainly by the EPS; chemical purity and crystallographic perfection (less contaminants or exogenous elements between the atoms chains which forms the crystal lattices); organization in chains (when consists of magnetofossils or fresh cells), uncommon shapes of particles (such as bullet-shaped or elongated crystals which can not be mimicked by inorganic processes); and a trendy to elongation of the crystals when they are organized in chains toward the crystallographic direction [111].

X-Ray Diffraction (XRD) is a potential technique to be used in the study of mineral biosignatures due to its capability of identifying crystalline phases such as inorganic and organic ordered structures preserved and/or secreted by living organisms. This kind of technique is selective and can identify synthetic or natural minerals (Tadic and Epple, 2004). Even if a material presents mixed crystal structures, the characteristic peaks produced from different planes of reflection may allow the identification of specific crystalline phases. Thus, XRD is a very important tool for evaluating the biogenicity of minerals (Che et al., 2016; Iñiguez et al., 2017), allowing the detection of past life evidence in ancient rocks, fossils and even rocks from Mars in the future.

The detection limit of XRD depends on the measurement geometry, incident photon energy, spot size, and flux. Compared to conventional X-ray diffractometers, synchrotron measurements allow the study of very dilute phases with high angular resolution (2θ 10^–4∘^), considering the Bragg-Brentano geometry and the measurement in 2θ, which can be used to deconvolute very close peaks. This can be used to distinguish biotic and abiotic crystals, for example, magnetic compounds like greigite and magnetites produced by bacteria (Miot et al., 2014; Till et al., 2017).

Still exploring the possibility of detecting biominerals, another technique applicable to biogenicity problems of minerals, the X-ray Magnetic Circular Dichroism (XMCD), is able to give information about the orbital magnetic moment and the spin of the material. The technique provides information about the 3d electronic states in transition metals, such as Fe, Ni, Co, etc., which is responsible for the magnetic properties of the minerals (Stöhr, 1999; Rogalev et al., 2006). XMCD has been increasingly used to provide detailed information about the electronic and magnetic structure of nanoparticles (van der Laan and Figueroa, 2014), and this can be interesting for the study of biogenicity, especially for the investigation of ferromagnetic biominerals, such as biogenic magnetite and greigite. The technique is based on the dichroic effect, which occurs when left and right polarized light passes through a material showing differences in the absorption coefficients. Dichroism can be caused by the spin or by the anisotropy of the material, such as the magnetic anisotropy of certain minerals (Stöhr, 1999). That is, depending on the crystallographic direction of the mineral, the absorption of the light will be different, generating different spectra. Bacteria can produce extracellular nanoparticles of magnetite by different metabolic pathways, such as the iron oxidation under aerobic conditions. Also, another group, the magnetotactic bacteria, can originate intracellular magnetite in chains, the magnetosomes. These biominerals can be part of the fossil record or can be part of rocks with dubious origin. The XMCD technique has been shown to be a good tool in some biogenicity problems, especially regarding ferromagnetic minerals, as it can provide information about the ratio of iron species in magnetites of different origins (biogenic and inorganic), crystallinity, mineralogical structure and purity of the crystal. The crystallographic and magnetic characteristics of biogenic magnetites that allows to differentiate them from the inorganic minerals (Thomas-Keprta et al., 2000), could also be detectable with XMCD. For example, Carvallo et al. (2008), combining data from TEM analysis, used XMCD to demonstrate the high purity and crystallinity of biogenic nanomagnetite, showing that these particles contained higher amount of Fe^2+^ than the abiogenic nanomagnetite. The authors utilized the technique to compare the ratio of Fe^2+^ and Fe^3+^ in biogenic and inorganic magnetite nanoparticles synthesized, concluding that biogenic ones have higher crystallinity and higher amount of Fe^2+^ when measured in comparison with the inorganic nanoparticles. The authors concluded that the difference signal between the biogenic and abiogenic XMCD spectra is bigger than any systematic instrumental error.

Also using XMCD, Coker et al. (2007) compared magnetosome crystals with extracellular magnetite and inorganic magnetite, showing the similarity of the magnetosomes with the stoichiometric magnetite and their higher chemical purity in comparison with the other non-intracellular crystals. The works of Coker et al. (2007) and Carvallo et al. (2008) can be useful by presenting parameters to differentiate biogenic and abiogenic magnetites, taking into account the high crystallinity and high Fe^2+^ content in the intracellular magnetites. The exceptional reducing power of bacteria such as *Shewanella putrefaciens* probably explains the high concentration of Fe^2+^ in comparison to nanoparticles of abiotic origin.

The optical activity from organic and inorganic compounds has also been proposed as a tool for the detection and characterization of biosignatures. For example, kerogen (Marshall et al., 2017; Shkolyar et al., 2018), proteins (Dartnell and Patel, 2014; Lin et al., 2015) and minerals can be associated with the past presence of life in an environment. Gaft et al. (2015) showed several minerals in which the optical activity may be associated with rocks formed in different contexts. The optical channels in these materials, i.e., the ions and/or defects responsible for the luminescence, are described as in function of oxidation state and their optical transition (wavelength emission, time decay) when stimulated with ultraviolet (UV), visible and infrared (IR) light. X-ray Excited Optical Luminescence (XEOL) can be used for the same purpose. However, the excitation with X-rays allows the observation of all optical active channels due to their capability of exciting core levels, making the optical process dependent of the lattice relaxation. Defects may be probed and explored, as well as their characteristics such as oxidation state, origin (intrinsically or extrinsically formed) (Teixeira et al., 2014; Finch et al., 2016; Rezende et al., 2016), and the environment they were formed, for example, when a living organism has started its fossilization process or even why a precious gemstone presents determined color (Tao, 2016).

XEOL is a photon-in/photon out technique in which X-rays are used to excite core levels and getting light emitted in the range from the UV to IR (Sham, 2002). It is site-selective and can be used with variable X-ray photon energy, deeply penetrating a structure to excite its optical channels to explore their origins. XEOL combined with techniques such as XRF and XRD can be a powerful tool to describe a whole picture about composition and elements distribution in natural materials. Beamlines of 4th generations synchrotrons, such as the Carnaúba beamline of the Sirius light source (Tolentino et al., 2017), in Brazil, will have specific setups for the application of multi-technique analysis (XRF, XAS, XRD, and XEOL) of environmental samples, such as rocks and fossils. It will be possible to map optical active channels with a micro/nanosized and high resolution probe that will allow to explore the presence, for example, of an ion inside of minerals from the bones of a fossil and their characteristics (Kolodny et al., 1996; Rakovan and Reeder, 1996).

## Conclusion

The complexity of attestations of biogenicity on geological and paleobiological materials makes it essential to explore multiple and complementary approaches in different length and sensitivity scales. For the micron- and nanoscales synchrotron-based techniques represent the forefront of the application of photons for the inspection of a wide range of materials, allowing complex and heterogeneous samples to be studied at an unprecedented level of detail. Synchrotron approaches are been consolidated as important tools for the deeper understanding of the records of ancient life on Earth and for the non-destructive investigation of extremely rare samples, such as meteorites and rocks that will be retrieved from Mars in the near-future sample return missions.

The recent developments in synchrotron sources also brings good perspectives for the study of biosignatures. The novel 4th generation sources, such as MAX IV in Sweden, Sirius in Brazil, and the upgraded sources ESRF-II in France, APS-U in the United States and Spring8-II in Japan are opening up new avenues for the nanoscale investigation of different types of materials. For geobiological specimens, these machines will allow the achievement of nanometric spatial resolution for resolving preserved morphological fossils and also microbial-mineral interactions with different chemical and morphological contrast information, high energy and high spectral resolution for probing, mapping and speciating heavy Z elements and high sensitivity to elements in trace concentrations. These advances will allow complex and important questions on the early chemical and morphological biosignatures to be attacked, likely consolidating synchrotron paleometry and nanopalebiology within biogeosciences and astrobiology.

## Author Contributions

All authors contributed to the literature revision and manuscript writing.

## Conflict of Interest

The authors declare that the research was conducted in the absence of any commercial or financial relationships that could be construed as a potential conflict of interest.

## References

[B1] Ade H. Urquhart S. G. (2002). “NEXAFS spectroscopy and microscopy of natural and synthetic polymers,” in *Chemical Applications of Synchrotron Radiation*, ed. Sham T. K. (River Edge, NJ: World Scientific Publishing), 285–355.

[B2] Alleon J. Bernard S. Guillou C. L. Carbonne J. M. Pont S. Beyssac O. (2016). Molecular preservation of 1.*88Ga* Gunflint organic microfossils as a function of temperature and mineralogy. *Nat. Commun.* 7:11977. 10.1038/ncomms11977 27312070 PMC4915024

[B3] Allwood A. C. Rosing M. T. Flannery D. T. Hurowitz J. A. Heirwegh C. M. (2018). Reassessing evidence of life in 3,700-million-year-old rocks of Greenland. *Nature* 563 241–244. 10.1038/s41586-018-0610-4 30333621

[B4] Banfield J. F. Moreau J. W. Chan C. S. Welch S. A. Little B. (2001). Mineralogical biosignatures and the search for life on Mars. *Astrobiology* 1 447–465. 10.1089/153110701753593856 12448978

[B5] Bazylinski D. A. Frankel R. B. Heywood B. R. Mann S. King J. W. Donaghay P. L. (1995). Controlled biomineralization of magnetite (Fe3O4) and greigite (Fe3S4) in a magnetotactic bacterium. *Appl. Environ. Microbiol.* 61 3232–3239. 16535116 10.1128/aem.61.9.3232-3239.1995 PMC1388570

[B6] Benning L. G. Phoenix V. R. Yee N. Tobin M. J. (2004). Molecular characterization of cyanobacterial silicification using synchrotron infrared micro-spectroscopy. *Geochim. Cosmochim. Acta* 68 729–741. 10.1016/s0016-7037(03)00489-7

[B7] Bernard S. Benzerara K. Beyssac O. Brown G. E. Jr. Grauvogel L. Stamm S. (2009). Ultrastructural and chemical study of modern and fossil sporoderms by scanning transmission X-ray microscopy (STXM). *Rev. Palaeobot. Palynol.* 156 248–261. 10.1016/j.revpalbo.2008.09.002

[B8] Bertrand L. Schoeder S. Anglos D. Breese M. Janssens K. Moini M. (2014). Mitigation strategies for radiation damage in the analysis of ancient materials. *Trends Anal. Chem.* 66 128–145. 10.1016/j.trac.2014.10.005

[B9] Brasier M. D. Antcliffe J. Saunders M. Wacey D. (2015). Changing the picture of Earth’s earliest fossils (3.5*–* 1.9 Ga) with new approaches and new discoveries. *Proc. Natl. Acad. Sci. U.S.A.* 112 4859–4864. 10.1073/pnas.1405338111 25901305 PMC4413290

[B10] Brocks J. J. Logan G. A. Buick R. Summons R. E. (1999). Archean molecular fossils and the early rise of eukaryotes. *Science* 285 1033–1036. 10.1126/science.285.5430.1033 10446042

[B11] Callefo F. Ricardi-Branco F. Hartmann G. A. Galante D. Rodrigues F. Maldanis L. (2019). Evaluating iron as a biomarker of rhythmites - an example from the last Paleozoic ice age of Gondwana. *Sediment. Geol.* 383 1–15. 10.1016/j.sedgeo.2019.02.002

[B12] Campbell K. A. Lyanne B. Y. Handley K. M. Jordan S. Farmer J. D. Guido D. M. (2015). Tracing biosignature preservation of geothermally silicified microbial textures into the geological record. *Astrobiology* 15 858–882. 10.1089/ast.2015.1307 26496526

[B13] Carvallo C. Sainctavit P. Arrio M. A. Menghy N. Wang Y. Nguema G. O. (2008). Biogenic vs. *abiogenic magnetite nanoparticles*: A XMCD study. *American Mineralogist* 93 880–885. 10.2138/am.2008.2713

[B14] Catling D. C. Krissansen-Totton J. Kiang N. Y. Crisp D. Robinson T. D. DasSarma S. (2018). Exoplanet biosignatures: a framework for their assessment. *Astrobiology* 18 709–738. 10.1089/ast.2017.1737 29676932 PMC6049621

[B15] Chang S. B. R. Kirschvink J. L. (1989). Magnetofossils, the magnetization of sediments, and the evolution of magnetite biomineralization. *Annual Review of Earth and Planetary Sciences* 17 169–195. 10.1146/annurev.earth.17.1.169

[B16] Che C. Parvez S. Glotch T. D. (2016). “Spectroscopic Study of Biosignatures in Clay-Rich Sediments: Implication for Martian Astrobiological Exploration,” in *Proceedings of the 47 Lunar and Planetary Science Conference*, (The Woodlands, TX).

[B17] Cockell C. S. (2014). Habitable worlds with no signs of life. *Philos Trans A Math Phys Eng Sci* 372 20130082. 10.1098/rsta.2013.0082 24664917 PMC3982426

[B18] Coker V. S. Pearce C. I. Lang C. van der Laan G. Patrick A. D. Telling N. D. (2007). Cation site occupancy of biogenic magnetite compared to polygenic ferrite spinels determined by X-ray magnetic circular dichroism. *European Journal of Mineralogy* 19 707–716. 10.1127/0935-1221/2007/0019-1758

[B19] Cunningham J. A. Thomas C. W. Bengtson S. Kearns S. L. Xiao S. Marone F. (2012). Distinguishing geology from biology in the Ediacaran Doushantuo biota relaxes constraints on the timing of the origin of bilaterians. *Proceedings of the Royal Society B: Biological Science* 279 2369–2376. 10.1098/rspb.2011.2280 22319125 PMC3350665

[B20] Cunningham J. A. Vargas K. Pengju L. Belivanova V. Marone F. Martínez-Pérez C. (2015). Critical appraisal of tubular putative eumetazoans from the Ediacaran Weng’an Doushantuo biota. *Proceedings of the Royal Society B: Biological Sciences* 282 20151169. 10.1098/rspb.2015.1169 26180072 PMC4528530

[B21] Dartnell L. R. Patel M. R. (2014). Degradation of microbial fluorescence biosignatures by solar ultraviolet radiation on Mars. *International Journal of Astrobiology* 13 112–123. 10.1017/s1473550413000335

[B22] De Gregorio B. T. Sharp T. G. Flynn G. J. Wirick S. Hervig R. L. (2009). Biogenic origin for Earth’s oldest putative microfossils. *Geology* 37 631–634. 10.1130/g25683a.1

[B23] Des Marais D. J. Allamandola L. J. Benner S. A. Boss A. P. Deamer D. Falkowski P. G. (2003). The NASA astrobiology roadmap. *Astrobiology* 3 219–235. 10.1089/153110703769016299 14577870

[B24] Des Marais D. J. Harwit M. O. Jucks K. W. Kasting J. F. Lin D. N. C. Lunine J. I. (2002). Remote sensing of planetary properties and biosignatures on extrasolar terrestrial planets. *Astrobiology* 2 153–181. 10.1089/15311070260192246 12469366

[B25] Des Marais D. J. Walter M. R. (1999). Astrobiology: exploring the origins, evolution, and distribution of life in the universe. *Annu Rev Ecol Syst* 30 397–420. 10.1146/annurev.ecolsys.30.1.397 11543275

[B26] Dodd M. S. Papineau D. Grenne T. Slack J. F. Rittner M. Pirajno F. (2017). Evidence for Early Life in Earth’s Oldest Hydrothermal Vent Precipitates. *Nature* 543 60–64. 10.1038/nature21377 28252057

[B27] Douglas S. Abbey W. Mielke R. Conrad P. Kanik I. (2008). Textural and mineralogical biosignatures in an unusual microbialite from Death Valley, California. *Icarus* 193 620–636. 10.1016/j.icarus.2007.08.019

[B28] Dove P. M. De Yoreo J. J. Weiner S. (2003). “Biomineralization,” in *Reviews in Mineralogy and Geochemistry*, (Washington, DC: Mineralogical Society of America), 54.

[B29] Dupraz C. Reid R. P. Braissant O. Decho A. W. Norman R. S. Visscher P. T. (2009). Processes of carbonate precipitation in modern microbial mats. *Earth Sci. Rev.* 96 141–162. 10.1016/j.earscirev.2008.10.005

[B30] Egli R. (2004). Characterization of individual rock magnetic components by analysis of remanence curves, 1. *unmixing natural sediments*. *Studia Geophysica et Geodaetica* 48 391–446. 10.1023/b:sgeg.0000020839.45304.6d

[B31] Finch A. A. Friis H. Maghrabi M. (2016). Defects in sodalite-group minerals determined from X-ray-induced luminescence. *Physics and Chemistry of Minerals* 43 481–491. 10.1007/s00269-016-0816-7

[B32] Gaboyer F. Milbeau C. L. Schwender P. Vannier P. Beblo-Vranesevic K. Rabbow E. (2017). Mineralization and Preservation of an extremotolerant Bacterium Isolated from an Early Mars Analog Environment. *Scientific Reports* 7 8775. 10.1038/s41598-017-08929-4 28821776 PMC5562696

[B33] Gaft M. Reisfeld R. Panczer G. (2015). *Modern Luminescence Spectroscopy of Minerals and Materials.* Cham: Springer International Publishing.

[B34] Garcia-Ruiz J. M. Hyde S. T. Carnerup A. M. Christy A. G. Van Kranendonk M. J. Welham N. J. (2003). Self assembled silica-carbonate structures and detection of ancient microfossils. *Science* 302 1194–1197. 10.1126/science.1090163 14615534

[B35] Golden D. C. Ming D. W. Schwandt C. S. Morris R. V. Yang S. V. Lofgren G. E. (2000). An experimental study on kinetically-driven precipitation of Ca-Mg-Fe carbonates from solution: implications for the low temperature formation of carbonates in Martian meteorite ALH84001. *Meteoritics and Planetary Sciences* 35 457–465. 10.1111/j.1945-5100.2000.tb01428.x

[B36] Gomes A. L. S. Becker-Kerber B. Osés G. L. Prado G. M. E. M. Kerber P. B. Barros G. B. B. (2019). Paleometry as a key tool to deal with paleobiological and astrobiological issues: some contributions and reflections on the Brazilian fossil record. *International Journal of Astrobiology* 19 1–15. 10.1017/s1473550418000538

[B37] Gorbushina A. A. Krumbein W. E. Volkmann M. (2002). Rock surfaces as life indicators: new ways to demonstrate life and traces on former life. *Astrobiology* 2 203–213. 10.1089/15311070260192273 12469369

[B38] Gordon P. R. Septhon M. A. (2016). Organic Matter Detection on Mars by Pyrolysis-FTIR: An Analysis of Sensitivity and Mineral Matrix Effects. *Astrobiology* 16 831–845. 10.1089/ast.2016.1485 27870586 PMC5124741

[B39] Grosch E. G. Muñoz M. Mathon O. McLoughlin N. (2017). “Earliest microbial trace fossils in Archaean pillow lavas under scrutiny: new micro-X-ray absorption near-edge spectroscopy, metamorphic and morphological constraints,” in *Earth System Evolution and Early Life: A Celebration of the Work of Martin Brasier*, Vol. 448 eds Brasier A. T. McIlroy D. McLoughlin N. (London: Geological Society), 57–70. 10.1144/sp448.8

[B40] Guido A. Mastandrea A. Tosti F. Demasi F. Blanco A. Elia M. D. (2012). Characterization of fossil organic matter with Fourier-Transform Infrared (FTIR) Spectroscopy: an attempt to record extraterrestrial life. *Memorie della Societa Astronomica Italiana Supplement* 20 64.

[B41] Guizar-Sicairos M. Boon J. J. Mader K. Diaz A. Menzel A. Bunk O. (2015). Quantitative interior x-ray nanotomography by a hybrid imaging technique. *Optica* 2 259–266.

[B42] Holler M. Diaz A. Guizar-Sicairos M. Karvinen P. Färm E. Härkönen E. (2014). X-ray ptychographic computed tomography at 16 nm isotropic 3D resolution. *Scientific Reports* 4 3857. 10.1038/srep03857 24457289 PMC3900995

[B43] Igisu M. Ueno Y. Takai K. (2018). FTIR microspectroscopy of carbonaceous matter in ∼ 3.5 *Ga seafloor hydrothermal deposits in the North Pole area, Western Australia*. *Progress in Earth and Planetary Science* 5 85.

[B44] Iñiguez E. Mendoza-Lavaniegos V. Kretzschmar T. G. (2017). Potential of hydrothermal altered rocks as potential scenario for search of biosignatures in exoplanets. *Procedia Earth and Planetary Science* 17 901–904. 10.1016/j.proeps.2017.01.012

[B45] Javaux E. J. (2019). Challenges in evidencing the earliest traces of life. *Nature* 572 451. 10.1038/s41586-019-1436-4 31435057

[B46] Javaux E. J. Knoll A. H. Walter M. R. (2001). Morphological and ecological complexity in early eukaryotic ecosystems. *Nature* 412 66–69. 10.1038/35083562 11452306

[B47] Jimenez-Lopez C. Romanek C. S. Bazylinski D. A. (2010). Magnetite as a prokaryotic biomarker: A review. *Journal of Geophysical Research* 115 G00G03.

[B48] Kolodny Y. Luz B. Sander M. Clemens W. A. (1996). Dinosaur bones: fossils or pseudomorphs? The pitfalls of physiology reconstruction from apatitic fossils. *Palaeogeography, Palaeoclimatology, Palaeoecology* 126 161–171. 10.1016/s0031-0182(96)00112-5

[B49] Konhauser K. O. Jones B. Phoenix V. R. Ferris G. Renaut R. W. (2004). The microbial role in hot spring silicification. *AMBIO: A Journal of the Human Environment* 33 552–558. 10.1579/0044-7447-33.8.552 15666688

[B50] Körnig A. Winklhofer M. Baumgartner J. Gonzalez T. P. Fratz P. Faivre D. (2014). Magnetite crystal orientation in magnetosome chains. *Advanced Functional Materials* 24 3926–3932. 10.1002/adfm.201303737 25866495 PMC4384753

[B52] Lawrence J. R. Swerhone G. D. W. Leppard G. G. Araki T. Zhang X. West M. M. (2003). Scanning Transmission X-Ray, Laser Scanning, and Transmission Electron Microscopy Mapping of the Exopolymeric Matrix of Microbial Biofilms. *Applied and Environmental Microbiology* 69 5543–5554. 10.1128/aem.69.9.5543-5554.2003 12957944 PMC194976

[B51] Lemelle L. Labrot P. Salomé M. Simionovici A. Viso M. Westall F. (2008). In situ imaging of organic sulfur in 700–800 My-old Neoproterozoic microfossils using X-ray spectromicroscopy at the S K-edge. *Org. Geochem.* 39 188–202. 10.1016/j.orggeochem.2007.10.008

[B53] Levin G. V. Straat P. A. (1977). Life on Mars? The Viking labeled release experiment. *Biosystems* 9 165–174. 10.1016/0303-2647(77)90026-0 20180

[B54] Lin S. Gao W. Tian Z. Yang C. Lu L. Mergny J. L. (2015). Luminescence switch-on detection of protein tyrosine kinase-7 using a G-quadruplex-selective probe. *Chemical Science* 7 4284–4290. 10.1039/c5sc01320h 29218197 PMC5707507

[B55] Lindsay J. F. Brasier M. D. McLoughlin N. Green O. R. Fogel M. Steele A. (2005). The problem of deep carbon - an Archean paradox. *Precambrian Research* 143 19–22.

[B56] Ma S. Boughton O. Karunaratne A. Jin A. Cobb J. Hansen U. (2016). Synchrotron imaging assessment of bone quality. *Clinical Reviews in Bone and Mineral Metabolism* 3 150–160. 10.1007/s12018-016-9223-3 27683260 PMC5018259

[B57] Maldanis L. Carvalho M. Almeida M. R. Freitas F. I. Andrade J. A. F. G. Nunes R. S. (2016). Heart Fossilization Is Possible and Informs the Evolution of Cardiac Outflow Tract in Vertebrates. *ELife* 5 1–12. 10.7554/eLife.14698 27090087 PMC4841765

[B58] Mann S. (2001). *Biomineralization: principles and concepts in bioinorganic materials chemistry.* Oxford: Oxford University Press, 198.

[B59] Manning-Berg A. R. Wood R. S. Williford K. H. Czaja A. D. Kah L. C. (2019). The taphonomy of proterozoic microbial mats and implications for early diagenetic silicification. *Geosciences* 9 40 10.3390/geosciences9010040

[B60] Marshall C. P. Oltcott M. A. Aitken J. B. Lai B. Vogt S. Breuer P. (2017). Imaging of Vanadium in Microfossils: A New Potential Biosignature. *Astrobiology* 17 1069–1076. 10.1089/ast.2017.1709 28910135

[B61] McKay D. S. Gibson E. K. Jr. Thomas-Keprta K. L. Vali H. Romanek C. S. Chillier X. D. F. (1996). Search for Past Life on Mars: Possible Relic Biogenic Activity in Martian Meteorite ALH84001. *Science* 273 924–930. 10.1126/science.273.5277.924 8688069

[B62] Meadows V. S. (2017). Reflections on O2 as a biosignature in exoplanetary atmospheres. *Astrobiology* 17 1022–1052. 10.1089/ast.2016.1578 28443722 PMC5655594

[B63] Meadows V. S. Reinhard C. T. Arney G. N. Parenteau M. N. Schwieterman E. W. Domagal-Goldman S. D. (2018). Exoplanet biosignatures: understanding oxygen as a biosignature in the context of its environment. *Astrobiology* 18 630–662. 10.1089/ast.2017.1727 29746149 PMC6014580

[B64] Miot J. Li J. Benzerara K. Sougrati M. T. Nguema G. O. Bernard S. (2014). Formation of single domain magnetite by green rust oxidation promoted by microbial anaerobic nitrate-dependent iron oxidation. *Geochimica et Cosmochimica Acta* 139 327–343. 10.1016/j.gca.2014.04.047

[B65] Mojzsis S. J. Arrhenius G. McKeegan K. D. Harrison T. M. Nutman A. P. Friend C. R. L. (1996). Evidence for life on Earth before 3.*800* million years ago. *Nature* 384 55–59. 10.1038/384055a0 8900275

[B66] Moussallam Y. Oppenheimer C. Scaillet B. Gaillard F. Kyle P. R. Peters N. (2014). Tracking the changing oxidation state of Erebus magmas, from mantle to surface, driven by magma ascent and degassing. *Earth and Planetary Science Letters, Elsevier* 393 200–209. 10.1016/j.epsl.2014.02.055

[B67] Newville M. (2014). Fundamentals of XAFS. *Reviews in Mineralogy and Geochemistry* 78 33–74. 10.2138/rmg.2014.78.2

[B68] Noffke N. (2015). Ancient sedimentary structures in the <3.7 *Ga Gillespie Lake Member, Mars, that resemble macroscopic morphology, spatial associations, and temporal succession in terrestrial microbialites*. *Astrobiology* 15 169–192. 10.1089/ast.2014.1218 25495393

[B69] Nutman A. P. Bennett V. C. Friend C. R. L. Kranendonk M. Chivas A. R. (2016). Rapid Emergence of Life Shown by Discovery of 3,700-Million-Year-Old Microbial Structures. *Nature* 537 535–538. 10.1038/nature19355 27580034

[B70] Ohtomo Y. Kakegawa T. Ishida A. Nagase T. Rosing M. T. (2014). Evidence for biogenic graphite in early Archaean Isua metasedimentary rocks. *Nature Geoscience* 7 25–28. 10.1038/ngeo2025

[B71] Osés G. L. Setembrino P. Voltani C. G. Prado G. M. E. M. Galante D. Rizzutto M. A. (2017). Deciphering pyritization-kerogenization gradient for fish soft-tissue preservation. *Scientific Reports* 7 1468. 10.1038/s41598-017-01563-0 28469235 PMC5431149

[B72] Perry R. S. Mcloughlin N. Lynne B. Y. Sephton M. A. Oliver J. D. Perry C. C. (2007). Defining biominerals and organominerals: Direct and indirect indicators of life. *Sedimentary Geology* 201 157–179. 10.1016/j.sedgeo.2007.05.014

[B73] Preston L. J. Melim L. Polyak V. J. Asmerom Y. Southam G. (2014). Infrared Spectroscopic Biosignatures from Hidden Cave, New Mexico: Possible Applications for Remote Life Detection. *Geomicrobiology Journal* 31 929–941. 10.1080/01490451.2014.913096

[B74] Rakovan J. Reeder R. J. (1996). Intracrystalline rare earth element distributions in apatite: Surface structural influences on incorporation during growth. *Geochimica et Cosmochimica Acta* 60 4435–4445. 10.1016/s0016-7037(96)00244-x

[B75] Rao D. V. Gigante D. E. Kumar Y. M. Ceasreo R. Bruneti A. Schiavon N. (2016). Synchrotron-based crystal structure, associated morphology of snail and bivalve shells by X-ray diffraction. *Radiation Physics and Chemistry* 127 155–164. 10.1016/j.radphyschem.2016.06.024

[B76] Rezende M. V. S. Montes P. J. R. Andrade A. B. Macedo Z. S. Valerio M. E. G. (2016). Mechanism of X-ray excited optical luminescence (XEOL) in europium doped BaAl2O4 phosphor. *Physical Chemistry Chemical Physics* 18 17646–17654. 10.1039/c6cp01183g 27306425

[B77] Rodelli D. Jovane L. Roberts A. P. Cypriano J. Abreu F. Lins U. (2018). Fingerprints of partial oxidation of biogenic magnetite from cultivated and natural marine magnetotactic bacteria using synchrotron radiation. *Environmental Microbiology Reports* 10 337–343. 10.1111/1758-2229.12644 29611897

[B78] Rogalev A. Wilhelm F. Jaouen N. Goulon J. Kappler J. P. (2006). “X-ray Magnetic Circular Dichroism: Historical Perspective and Recent Highlights,” in *Magnetism: A Synchrotron Radiation Approach, Lecture Notes in Physics*, eds Beaurepaire E. Bulou H. Scheurer F. Kappler J. P. (Heidelberg: Springer), 697.

[B79] Sancho-Tomás M. Somogyi A. Medjoubi K. Bergamaschi A. Visscher P. T. Van Driessche A. E. S. (2018). Distribution, redox state and (bio)geochemical implications of arsenic in present day microbialites of Laguna Brava, Salar de Atacama. *Chemical Geology* 490 13–21. 10.1016/j.chemgeo.2018.04.029

[B80] Schopf J. W. (1993). Microfossils of the Early Archean Apex chert: new evidence of the antiquity of life. *Science* 260 640–646. 10.1126/science.260.5108.640 11539831

[B81] Schopf J. W. Kitajima K. Spicuzza M. J. Kudryavtsev A. B. Valley J. W. (2018). SIMS Analyses of the Oldest Known Assemblage of Microfossils Document Their Taxon-Correlated Carbon Isotope Compositions. *Proceedings of the National Academy of Sciences* 115 53–58. 10.1073/pnas.1718063115 29255053 PMC5776830

[B82] Schwieterman E. W. Kiang N. Y. Parenteau M. N. Harman C. E. DasSarma S. Fisher T. M. (2018). Exoplanet Biosignatures: A Review of Remotely Detectable Signs of Life. *Astrobiology* 18 663–708. 10.1089/ast.2017.1729 29727196 PMC6016574

[B83] Sforna M. C. Daye M. Phillippot P. Somogyi A. van Zuilen M. A. Medjoubi K. (2016). Patterns of metal distribution in hypersaline microbialites during early diagenesis: Implications for the fossil record. *Geobiology* 15 259–279. 10.1111/gbi.12218 27935656

[B84] Sham T.-K. (2002). *Chemical Applications of Synchrotron Radiation*, Vol. 12 Singapore: World Scientific.

[B85] Shkolyar S. Eshelman E. J. Farmer J. D. Hamilton D. Daly M. G. Youngbull C. (2018). Detecting Kerogen as a Biosignature Using Colocated UV Time-Gated Raman and Fluorescence Spectroscopy. *Astrobiology* 18 431–453. 10.1089/ast.2017.1716 29624103

[B86] Steele A. McGubbin F. M. Fries M. Kater L. Boctor N. Z. Fogel M. L. (2012). A reduced organic carbon component to Martian Basalts. *Science* 337 212–215. 10.1126/science.1220715 22628557

[B87] Stevens A. H. McDonald A. Koning C. Riedo A. Preston L. J. Ehrenfreund P. (2019). Detectability of biosignatures in a low-biomass simulation of martian sediments. *Scientific Reports* 9 9706. 10.1038/s41598-019-46239-z 31273294 PMC6609699

[B88] Stöhr J. (1999). Exploring the microscopic origin of magnetic anisotropies with X-ray magnetic circular dichroism (XMCD) spectroscopy. *J. Magn. Magn. Mater.* 200 470–497. 10.1016/s0304-8853(99)00407-2

[B89] Sugitani K. Mimura K. Takeuchi M. Yamaguchi T. Suzuki K. Senda R. (2015). A Paleoarchean coastal hydrothermal field inhabited by diverse microbial communities: The Strelley Pool Formation, Pilbara Craton, Western Australia. *Geobiology* 13 522–545. 10.1111/gbi.12150 26189535

[B90] Summons R. E. Albrecht P. McDonald G. Moldowan J. M. (2008). Molecular biosignatures. *Space Science Reviews* 135 115–132.

[B91] Tadic D. Epple M. (2004). A thorough physicochemical characterisation of 14 calcium phosphate-based bone substitution materials in comparison to natural bone. *Biomaterials* 25 987–994. 10.1016/s0142-9612(03)00621-5 14615163

[B92] Tafforeau P. Boistel R. Boller E. Bravin A. Brunet M. Chaimanee Y. (2006). Applications of X-Ray Synchrotron Microtomography for Non-Destructive 3D Studies of Paleontological Specimen. *Applied Physics A* 83 195–202. 10.1007/s00339-006-3507-2

[B93] Tao H. (2016). “The Applications of Ultraviolet Visible Absorption Spectrum Detection Technology in Gemstone Identification,” in *Proceedings of the 5th International Conference on Materials Engineering and Advanced Technologies (ICMEAT).” DEStech Transactions on Materials Science and Engineering Meet*, (Quebec City).

[B94] Teixeira V. C. Montes P. J. R. Valerio M. E. G. (2014). Structural and optical characterizations of Ca2Al2SiO7: Ce3+, Mn2+ nanoparticles produced via a hybrid route. *Optical Materials* 36 1580–1590. 10.1002/bio.3025 26394791

[B95] Thomas-Keprta K. L. Bazylinski D. A. Kirschvink J. L. Clemett S. J. Mckay D. S. Wentworth S. J. (2000). Elongated prismatic magnetite crystals in ALH84001 carbonate globules: Potential Martian magnetofossils. *Geochimica et Cosmochimica Acta* 64 4049–4081. 10.1016/s0016-7037(00)00481-6 11543573

[B96] Thomas-Keprta K. L. Clemett S. J. Bazylinski D. A. Kirschvink J. L. McKay D. S. Wentworth S. J. (2002). Magnetofossils from ancient Mars: a robust biosignature in the martian meteorite ALH84001. *Applied and Environmental Microbiology* 68 3663–3672. 10.1128/aem.68.8.3663-3672.2002 12147458 PMC123990

[B97] Thomas-Keprta K. L. Clemett S. J. McKay D. S. Gibson E. K. Jr. Wentworth S. J. (2009). Origins of magnetite nanocrystals in Martian meteorite ALH84001. *Geochimica et Cosmochima Acta* 73 6631–6677. 10.1016/j.gca.2009.05.064

[B98] Till J. Guyodo Y. Lagroix F. Morin G. Menguy N. Nguema G. O. (2017). Presumed magnetic biosignatures observed in magnetite derived from abiotic reductive alteration of nano goethite. *Comptes Rendus Géoscience* 349 63–70. 10.1016/j.crte.2017.02.001

[B99] Tolentino H. C. N. Soares M. M. Perez C. A. Vicentin F. C. Abdala D. B. Galante D. (2017). Carnaúba: the coherent x-ray nanoprobe beamline for the Brazilian Synchrotron Sirius/LNLS. *Journal of Physics: Conference Series* 849 012057 10.1088/1742-6596/849/1/012057

[B100] van der Laan G. Figueroa A. I. (2014). X-ray magnetic dichroism – A versatile tool to study magnetism. *Coord. Chem. Rev.* 277-278 95–129. 10.1016/j.ccr.2014.03.018

[B101] van Zuilen M. Lepland A. Arrhenius G. (2002). Reassessing the evidence for the earliests traces of life. *Nature* 418 627–630. 10.1038/nature00934 12167858

[B102] Wacey D. Kilburn M. R. Saunders M. Cliff J. Brasier M. D. (2011). Microfossils of sulphur-metabolizing cells in 3.4*-billion-year-old rocks of Western Australia*. *Nature Geosciences* 4 698 10.1038/ngeo1238

[B103] Wacey D. Menon S. Green L. Gerstmann D. Kong C. Mcloughlin N. (2012). Taphonomy of very ancient microfossils from the 3400Ma Strelley Pool Formation and 1900Ma Gunflint Formation: New insights using a focused ion beam. *Precambrian Research.* 220–221 234–250. 10.1016/j.precamres.2012.08.005

[B104] Walker S. I. Bains W. Cronin L. DasSarma S. Danielache S. Domagal-Goldman S. (2018). Exoplanet biosignatures: future directions. *Astrobiology* 18 779–824. 10.1089/ast.2017.1738 29938538 PMC6016573

[B105] Walsh M. M. (1992). Microfossils and possible microfossils from the Early Archean Onverwacht Group, Barberton Mountain Land, South Africa. *Precambrian Research* 54 271–293. 10.1016/0301-9268(92)90074-x 11540926

[B106] Westall F. (2008). Morphological biosignatures in terrestrial and extraterrestrial materials. *Space Science Reviews* 135 95–114. 10.1007/978-0-387-77516-6_9

[B107] Westall F. (2009). Life on an anaerobic planet. *Science* 232 471–472. 10.1126/science.1167220 19164738

[B108] Westall F. Cavalazzi B. (2011). “Biosignatures in Rocks,” in *Encyclopedia of Geobiology. Encyclopedia of Earth Sciences Series*, eds Reitner J. Thiel V. (Dordrecht: Springer).

[B109] Westall F. Hickmann-Lewis K. Cavalazzi B. (2019). “Biosignatures in deep time,” in *Biosignatures for Astrobiology, Advances in Astrobiology and Biogeophysics*, eds Cavalazzi B. Westall F. (Cham: Springer Nature Switzerland AG).

[B110] Westall F. Steele A. Toporski J. Walsh M. Allen C. Guidry S. (2000). Polymeric substances and biofilms as biomarkers in terrestrial materials: implications for extraterrestrial samples. *Journal of Geophysical Research* 105 24511–24527. 10.1029/2000je001250

[B111] White B. (1974). Microfossils from the Late Precambrian Altyn Formation of Montana. *Nature* 247 452–453. 10.1038/247452a0

[B112] Williams R. P. J. Frausto da Silva J. J. R. (1996). *The natural selection of the chemical elements.* Clarendon, TX: Oxford, 646.

