# Corrigendum: Evaluating Biogenicity on the Geological Record With Synchrotron-Based Techniques

**DOI:** 10.3389/fmicb.2019.02901

**Published:** 2019-12-17

**Authors:** Flavia Callefo, Lara Maldanis, Verônica C. Teixeira, Rodrigo Adrián de Oliveira Abans, Thiago Monfredini, Fabio Rodrigues, Douglas Galante

**Affiliations:** ^1^Brazilian Synchrotron Light Laboratory, Brazilian Center for Research in Energy and Materials, Campinas, Brazil; ^2^Institute of Physics of São Carlos, University of São Paulo, São Paulo, Brazil; ^3^Interunits Graduate Program in Biotechnology, University of São Paulo, São Paulo, Brazil; ^4^Fundamental Chemistry Department, University of São Paulo, São Paulo, Brazil

**Keywords:** synchrotron, biogenicity, spectroscopy, imaging, biostructures

In the original article, the name of one of the authors was incorrectly spelled in the reference Lemelle et al., 2008. It was spelled Lamelle et al., 2008 but should be Lemelle et al., 2008.

The authors apologize for this error and state that this does not change the scientific conclusions of the article in any way. The original article has been updated.

